# Galactose alters early-life development and exerts sex-specific nutritional programming effects on lifespan in *Drosophila melanogaster*

**DOI:** 10.3389/fnut.2026.1809195

**Published:** 2026-04-30

**Authors:** Peixin Sun, Shiying Shao, Steven M. Driever, Deli Zhang, Jaap Keijer, Evert M. van Schothorst

**Affiliations:** 1 Human and Animal Physiology, Wageningen University and Research, Wageningen, Netherlands; 2 Centre for Crop Systems Analysis, Wageningen University and Research, Wageningen, Netherlands

**Keywords:** dietary carbohydrates, DOHAD, growth & development, longevity, metabolic programming, oxygen consumption

## Abstract

**Background:**

Nutritional programming by early-life nutrition exerts long-lasting effects on later-life health. In mammals, galactose, as a component of the milk sugar lactose, is consumed during lactation. Mouse studies suggest that prolonged postweaning galactose consumption improves later-life metabolic health, but it is unclear whether these benefits translate into increased lifespan. The fruit fly *Drosophila melanogaster* is an attractive model for studying programming effects on lifespan due to its relatively short lifespan.

**Methods:**

*Drosophila* larvae were fed on either a galactose- or standard glucose-based diet. Upon eclosion, adult male and female flies were switched to either a standard or a high-glucose obesogenic diet. We assayed larval developmental time and pupal metabolism, followed by adult body weight, triacylglycerol (TAG) levels, and lifespan.

**Results:**

In early-life, galactose consumption significantly prolonged larval development time, increased the pupal volume, reduced the pupal mitochondrial mass, and increased the energy expenditure of pupae in a body-weight-dependent manner. In adults fed a glucose diet, early-life galactose consumption significantly increased body weight, decreased whole-body TAG content, and reduced survival rate only in female flies, leaving males unaffected. These programming effects were absent when the adult flies were fed a high-glucose obesogenic diet.

**Conclusion:**

We newly show that dietary galactose has significant effects on early-life development of *Drosophila*, with sexual dimorphism in nutritional programming effects, which depend on the later-life diet.

## 1 Introduction

Nutritional programming by early-life nutrition exerts long-lasting effects on later-life health (1–4). Most nutritional programming studies have focused on the effects of malnutrition. For instance, exposure to famine during gestation is associated with a higher risk of insulin resistance in 50-year-old men and women (5), and maternal obesogenic high-fat diet intake gave long-lasting detrimental effects on the metabolic health of offspring in a mouse model (6, 7). In contrast, protein restriction in post-natal early-life extends lifespan in the mouse (8) and fruit fly (9) models, and shows protective effects on obesity risk in humans (10, 11). Early-life carbohydrate restriction has been linked to a lower risk of type 2 diabetes in later-life in humans (12). These studies highlight the importance of early life macronutrient intake. However, the type of nutrients consumed, not just the quantity, is another crucial factor that is often overlooked. Different types of carbohydrates have clearly different metabolic effects (13, 14). However, the long-term health effects of different types of carbohydrates, in particular the simple sugars like dietary mono- and disaccharides, in early-life remain poorly understood.

Galactose is one of the three main dietary monosaccharides, next to glucose and fructose. Galactose has been used to induce aging models (15), however, as a component of the milk sugar lactose, it plays a crucial role in early postnatal development, and emerging evidence suggests it may offer beneficial health effects (16). For instance, in adult humans, galactose intake promoted fat mobilization compared to glucose (17) and galactose consumption improves insulin sensitivity in rodents compared to glucose (18). Given the beneficial health effects of extended breastmilk feeding (19–21), the World Health Organization (WHO) propagates extended breastfeeding beyond the age of 2 years to optimally provide beneficial health effects to the offspring. It is tempting to speculate that galactose intake partly contributes to the observed beneficial effects of extended breastfeeding, since lactose is the main carbohydrate component of milk and a principal source of galactose, and galactose consumption drops significantly after weaning. Therefore, it may be hypothesized that galactose consumption in early-life has long-lasting beneficial effects on later-life health. This is supported by the observation in a mouse model that prolonged consumption of a lactose-mimic (isocaloric, free galactose + glucose), for 3 weeks immediately after weaning, showed long-term beneficial effects in females, such as attenuated obesity, after subsequent 9 weeks on an obesogenic diet (22). Another mouse study showed that the postweaning galactose intervention did not decrease fat mass but improved circulating adiponectin levels in females, after subsequent 9 weeks on a more harmful obesogenic diet (23). Adiponectin is an insulin-sensitizing hormone with higher levels being associated with better metabolic health (24). Therefore, the programming effects of galactose on later-life health are potentially promising. These latter two mouse studies (22, 23) focused on the obesity -related effects until a relatively young (adult) stage of 15 weeks, and it is unclear whether galactose consumption can have life-long effects, which deserve further investigation. In this regard, the fruit fly *Drosophila melanogaster* (*Drosophila*) has added value because of its substantially shorter lifespan of around 90 days, compared to over 3 years for a laboratory mouse, such as C57BL/6J (25). *Drosophila* has been used for nutritional and metabolic disease studies (26, 27), including nutritional programming studies (28, 29). Moreover, *Drosophila* shares common metabolic pathways with mammals. Carbohydrate catabolic pathways, including the Leloir pathway metabolizing galactose, are also present in *Drosophila* (30). Galactose is also a physiologically relevant carbohydrate in *Drosophila* and is mainly metabolized in the fat body (31). The *Drosophila* fat body is considered the combination of mammalian adipose tissue and liver and is the key storage depot for triacylglycerol (TAG), the major storage form of energy. Of note, a galactose-containing postweaning diet reduced hepatic TAG content in the mouse model (32) and a galactose-containing drink enhanced endogenous fat mobilization and oxidation in healthy women with obesity (17). It is unclear whether galactose affects TAG content in *Drosophila* as well.

To investigate the long term nutritional programming effects by galactose at physiological dietary relevant levels, we studied the effects of 5% (w/v) galactose or glucose on development in early-life, and on adult life in the context of a 5% w/v glucose diet or an obesogenic 20% w/v glucose diet, and examined how this translated into fat storage and effects on lifespan.

## 2 Materials and methods

### 2.1 *Drosophila* stock and diet preparation

The stock *Drosophila melanogaster* (W^1118^ strain) were housed in vials (789008, Kisker Biotech, Steinfurt, Germany), with a density of 20 flies per vial with a mixture of males and females, and were maintained at a standard temperature of 25 °C, a 12-h light-dark cycle.

Diet preparation is based on protocols previously described (33). The composition of the diets is shown in Table 1, and they were used as follows. During the larval period, larvae were fed a standard control diet containing glucose (GLU) or a diet containing galactose (GAL). During the adult period, the flies were fed a GLU or a high-glucose (HGLU) diet. Groups were termed according to the diets using the naming convention early diet-later diet (e.g., GLU-GLU means *Drosophila* fed GLU diet during larval period and GLU diet in adulthood). The stock flies were maintained on the GLU diet.

**Table 1:** Dietary composition^a^.

Ingredients	GLU	GAL	HGLU
Glucose (M)	0.28	0	1.12
Galactose (M)	0	0.28	0
Yeast % (w/v)	10	10	10
Agar % (w/v)	1.5	1.5	1.5
Nipagin % (w/v)	0.13	0.13	0.13
Propionic acid % (v/v)	0.3	0.3	0.3

^*a*^Glucose (G7021, Merck, Amsterdam, The Netherlands); galactose (G0750, Merck); Dry yeast (7502231540858, De Zuidmolen, Groesbeek, The Netherlands); agar (76050048, Boom, Meppel, The Netherlands); nipagin (H3647, Merck); propionic acid (P1386, Merck).

### 2.2 Synchronized egg collection

For each experiment, synchronized eggs were used to ensure all eggs were from the same parental population, with closely matched hatch time (34). Approximately 400 adult flies (approximately sex ratio 1:1), a mixture of males and females aged 4 to 12 days post-eclosion, were placed in an embryo collection cage (size: 8.75 cm x 14.8 cm; 59–101, Genesee Scientific, San Diego, USA). The cage was closed containing a Petri dish with 5% glucose diet and yeast paste (a mixture of water and yeast, to stimulate the egg production) for a 24-h acclimatization period. Following this, the Petri dish was replaced by a new one with fresh 5% glucose diet and yeast paste, and the flies were allowed to mate and produce synchronized eggs over a 5-h period. Eggs were gently collected using a phosphate-buffered saline (PBS) wash and a cut pipette tip and then seeded at a density of 80 to 120 eggs per 6 ml of diet per vial. Following this protocol, approximately 1,200 eggs can be obtained per collection cage. Depending on the experimental requirements, multiple collection cages were prepared to ensure sufficient numbers of pupae or flies.

### 2.3 Larval developmental time and pupal size

Following egg seeding on either 5% glucose or 5% galactose diets, eggs in each vial (each representing a biological replicate) were counted under a stereo microscope (M80, Leica microsystems, Wetzlar, Germany). Pilot studies were first performed to estimate when pupation begins, and the timing of egg seeding was adjusted accordingly to ensure that most pupation events occurred during daytime hours. For the real experiment, the time to pupation was recorded every 5 h during the daytime (07:30–18:30h). The ratio of pupae to original eggs was used to generate early-life developmental curves, which fit a four-parameter logistic curve, from which the time that 50% of eggs formed pupae was interpolated. The maximum ratio indicated the survival rate during the larval period (assayed in an independent experiment). Pupae were gently removed from vials with a brush within 1 h after pupae formation, placed on graph paper, and photographed under a microscope. Pupal length and width were measured individually using ImageJ software. The pupal volume was calculated based on the published equation: $Pupal\;volume=(\frac{4}{3})\pi (\frac{L}{2}){\left(\frac{D}{2}\right)}^{2}\;$(L, length; D, diameter) (35). The pupal volume of 20 collected pupae from the same vial was averaged into a single value.

### 2.4 Pupal oxygen consumption

Oxygen consumption as a proxy for energy expenditure by pupae was measured using a differential O_2_ analyzer system (Qubit Systems, Kingston, Canada) with two parallel 10 ml insect-specific chambers, of which one chamber was used as the reference chamber and remained empty. Twenty-five pupae formed within 1 h were placed in the other chamber. The oxygen concentration difference (delta concentration) between the two chambers was recorded every second. The oxygen consumption for each sample was measured over a 20-min period to reach a stable delta O_2_ concentration. The oxygen consumption of an average individual pupa was calculated based on the following formula:

$Oxygen\;consumption\;\left(\mu l/\min\right)=\frac{Delta\;concentration\;\times \;S}{N}\;$(S: airflow (μl/min), N: pupa number). The body weight of the pupae was measured afterwards by an analytical balance (30355500, Mettler Toledo, Tiel, The Netherlands). This procedure was repeated 8 times in the GLU group and 10 times in the GAL group with different batches of pupae.

### 2.5 Citrate synthase assay

Synchronized eggs were seeded on either 5% glucose or 5% galactose diets (8 vials per dietary group), with each vial treated as one biological replicate. Five pupae per vial were collected immediately after pupation and were snap frozen in liquid nitrogen. The citrate synthase content as a mitochondrial mass marker was assayed according to the manufacturer's instructions (CS0720, Merck). The collected pupae were then homogenized in 150 μl lysis buffer containing 50 mM Tris-HCl pH 7.4, 150 mM NaCl, 1% Triton X-100, 1% glycerol,1 mM ethylenediaminetetraacetic acid, 2 μM trichostatin A, 10 mM nicotinamide and one tablet per 10 ml of both protease (4693132001, Merck) and phosphatase inhibitor cocktail (4906845001, Merck). The homogenates were then submitted to a freeze–thaw cycle, after which the samples were centrifuged at 10,000 g and 4 °C to remove cell debris. The protein content was determined using the DC (detergent compatible) protein assay reagent (5000116, Bio-Rad, Veenendaal, The Netherlands). Samples, acetyl coenzyme A, and 5,5'-Dithiobis-(2-nitrobenzoic acid) were first added to measure the baseline absorbance at 412 nm. Next, oxaloacetate was added to initiate the reaction, and changes in absorbance were measured every 30 s for 20 min using a Synergy HT Multi-detection microplate reader (BioTek Instruments, Inc., Winooski, USA). The citrate synthase content was then calculated using the equation according to the manufacturer's instructions: $Citrate\;synthase\;content\;\left(\mu mol/min/mg\;protein\right)=\;\frac{Delta\;A412\;\times \;V}{\varepsilon \;\times \;L\;\times \;P}\;$with parameters Delta A412: the changes in absorbance after 1.5 min of the reaction at 412 nm; V: reaction volume, 0.2 (ml); ε: 13.6 (mM^−1^ cm^−1^), the extinction coefficient of 5,5′-Dithiobis-(2-nitrobenzoic acid) at 412 nm; L: the pathlength, 0.552 (cm); P: the protein input (mg).

### 2.6 Body weight and whole-body TAG measurement of flies

Synchronized eggs were seeded on GLU or GAL diets (12 vials per dietary condition), with each vial treated as one biological replicate. Groups of 10 to 20 flies were first anesthetized by CO_2_, and total body weight was measured and then averaged to estimate the individual body weight. Four random flies per vial (8 vials per group) were homogenized in 150 (for males) or 300 μl (for females) 0.05% PBST (PBS with 0.05% Tween 20), and the homogenate was aliquoted for either whole-body TAG or protein measurements. The homogenate for whole-body TAG content measurement was heated at 65 °C for 5 min using TAG assay kit reagents (10720P, Instruchemie, Delfzijl, The Netherlands), as previously published (36). The homogenate for protein measurement was 5 times diluted and the protein content was measured by the DC protein assay reagent (Bio-Rad) according to the manual. TAG and protein assays were performed in duplicates, and the samples with a coefficient of variation (CV%) higher than 10% were excluded from analyses. The whole-body TAG content was normalized by the protein content.

### 2.7 Lifespan assay

Synchronized eggs were seeded onto either 5% glucose or galactose diets. Day 0 was defined at eclosion, and both male and female flies were transferred to an embryo collection cage with the 5% glucose diet for 2 days to ensure sexual maturation at a density of about 400 flies per cage. Male and female flies were then separated under CO_2_ anesthesia and transferred to either 5% glucose or 20% glucose diet, at a density of 20 flies per vial (*n* = 7 vials per group for flies fed 5% glucose diet; *n*=6 for male flies fed 20% glucose diet; *n* = 5 for female flies fed 20% glucose diet). The diet was replaced three times a week, and the mortality was recorded daily as published (34).

### 2.8 Statistical analysis

The developmental curve was analyzed by two-way ANOVA. Student's *t*-test was used for 50% developmental time, survival rate, pupal volume, absolute oxygen consumption, and citrate synthase content when the data passed normality or lognormality test, otherwise, a Mann Whitney test was used. Data were statistically analyzed using Prism (Version 10.4.1, GraphPad Software Inc., San Diego, USA), unless stated otherwise. The oxygen consumption was plotted versus body weight and was analyzed using ANCOVA with body weight as a covariate in R 4.4.2, following the approach as described (37, 38). Normalized oxygen consumption was calculated at a constant body weight using the regression lines from the plot of oxygen consumption versus body weight per group, following the approach described before (38). Adult body weight and whole-body TAG content were analyzed by two-way ANOVA within each timeslot and the Šidák test was performed within each sex if residuals passed the Shapiro-Wilk normality test. If residuals did not pass the Shapiro-Wilk normality test, data were analyzed by Generalized Linear Model with Gamma distribution and log link in R 4.4.2. Survival curves were compared using the log-rank (Mantel–Cox) test. Hazard ratios and 95% confidence intervals were estimated using a log-rank–based method as implemented in GraphPad Prism. The median survival was defined as the age at which 50% of the population remained alive. The maximum survival referred to the mean lifespan of the longest-lived 10% of individual flies. The average lifespan represented the mean lifespan across the entire population. Data is shown as mean ± SD. A trend is noted when 0.05 < *P* < 0.1. Significance is set at *P* < 0.05 and shown as follows: ^*^ *P* < 0.05, ^**^ *P* < 0.01, ^***^ *P* < 0.001, ^****^ *P* < 0.0001.

## 3 Results

### 3.1 Developmental time and survival rate during the larval period

**Figure 1:**
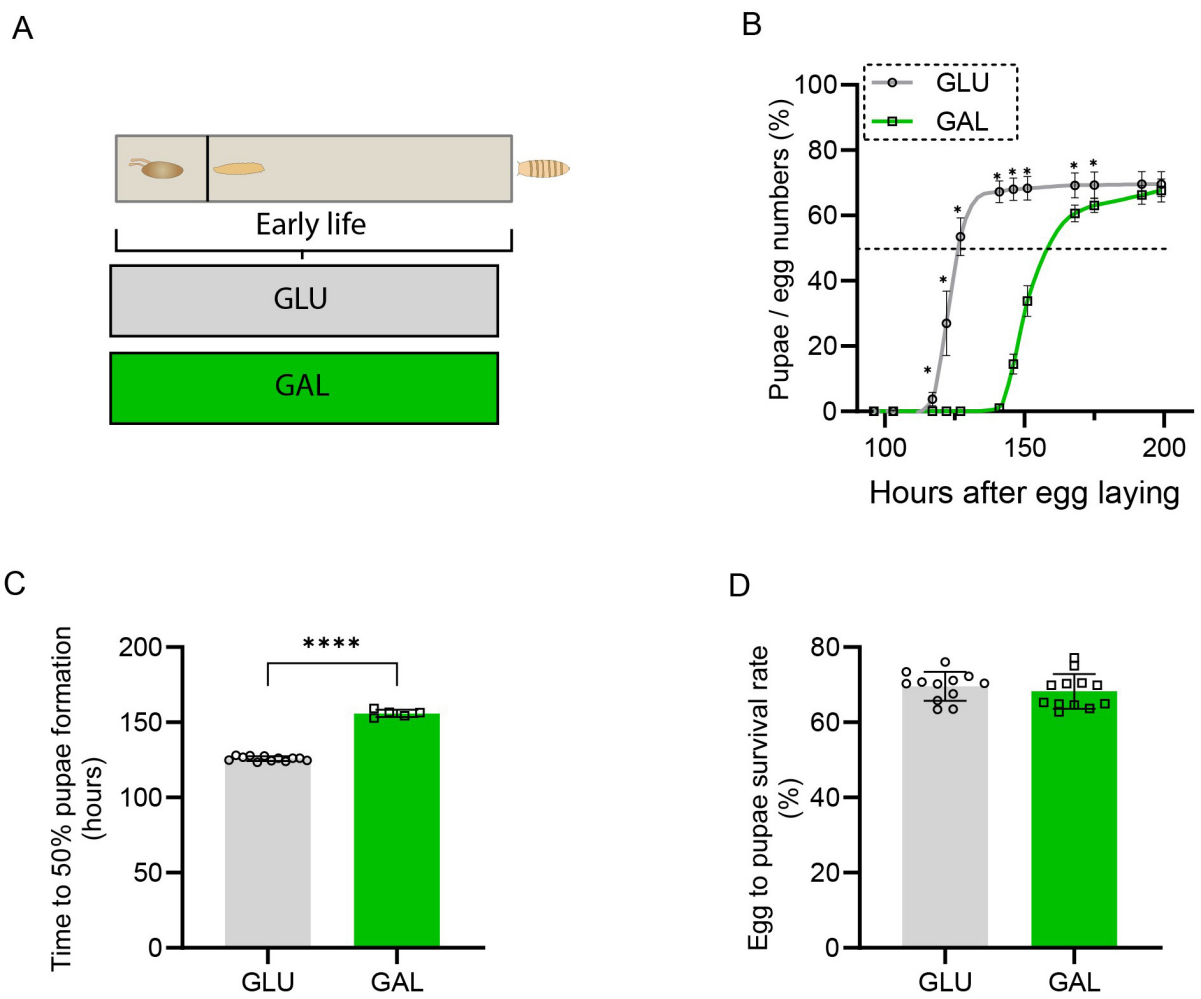
Galactose extended developmental time in the larval period without affecting survival rate. **(A)** Early-life experimental scheme. Eggs are seeded, followed by the larval stage when they eat, which stops once they become pupae. **(B)** Pupae formation in time (*n* = 12 replicates (vials) in GLU, *n* = 5 replicates in GAL, with 80-120 eggs per replicate). **(C)** Developmental time from egg laying until 50% of the eggs have pupated; values represent 50% pupation time per sample (*n* = 12 replicates (vials) in GLU, *n* = 5 replicates in GAL, with 80-120 eggs per replicate). **(D)** Egg to pupae survival rate, which is the percentage of numbers of total pupae formed out of the numbers of seeded eggs; values represent the maximum survival rate per sample (*n* = 12 replicates (vials) per group, with 80-120 eggs per replicate). Statistical analysis was performed by two-way ANOVA for panel B and by Student's *t*-test for panels C and D (*P < 0.05; ****P < 0.0001). The unequal sample sizes in panels B and C result from technical loss.

Feeding the developmental diets (galactose versus glucose) during the larval period (Design scheme is shown in Figure 1A), the only period when fruit flies consume food until they fully eclose, resulted in a significantly longer time to pupa formation in GAL compared to GLU (Figures 1B, C), and the eclosion time was also accordingly extended in GAL. The ratio of the total number of pupae formed to the initial number of eggs seeded was not different, suggesting that the survival rate during the larval period remained unaffected (Figure 1D). Meanwhile, the survival rate during the pupal period was also not different between groups, with 91.1 ± 3.5% of pupae from GAL versus 92.3 ± 2.7 % of pupae from GLU successfully eclosing as adults.

### 3.2 Pupal volume and oxygen consumption

**Figure 2:**
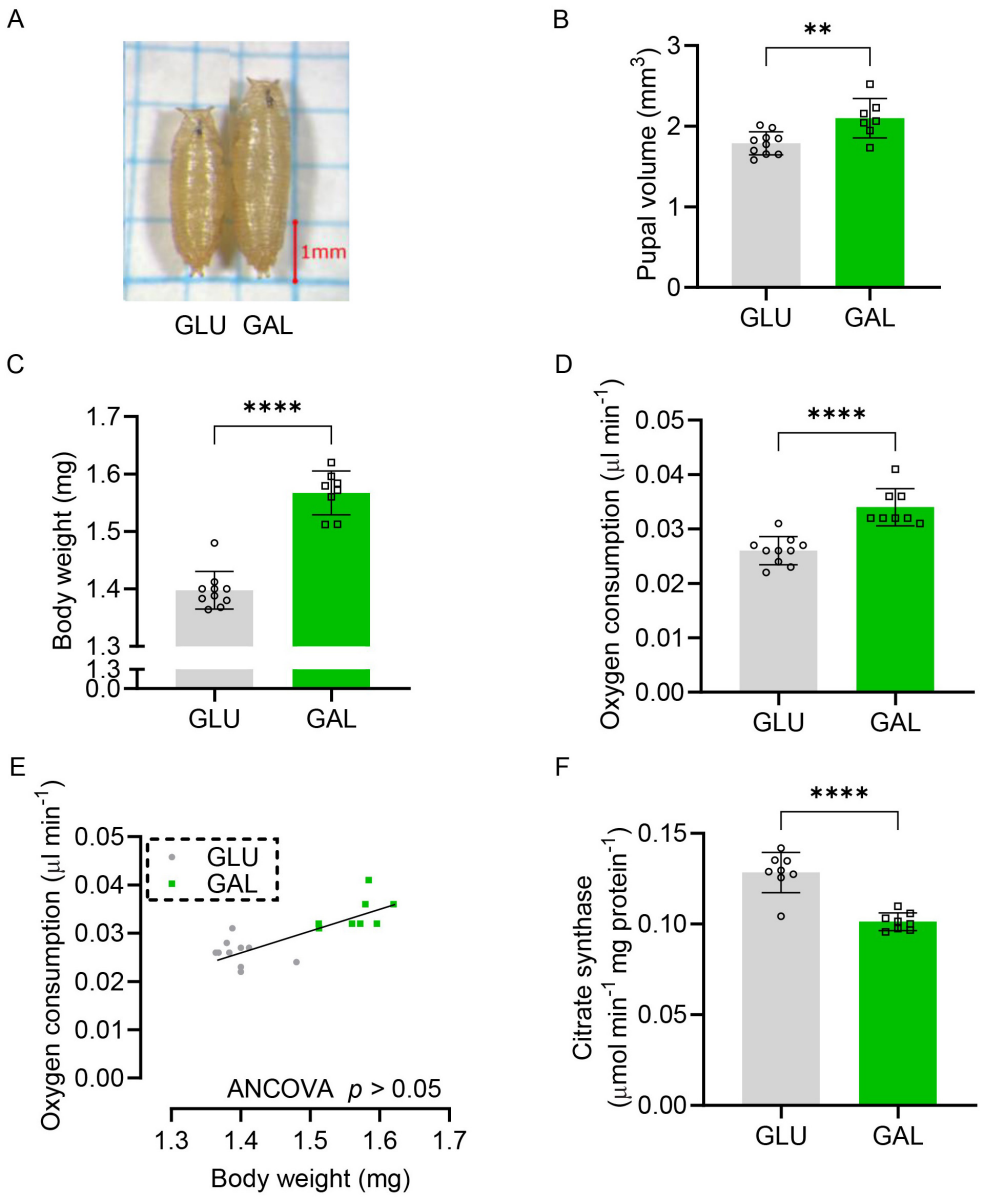
Galactose increased pupal size and pupal oxygen consumption but reduced the mitochondrial mass. **(A)** Representative image of pupae formed from larvae grown on either 5% glucose (GLU) or 5% galactose (GAL) diets. **(B)** Pupal volume after pupae formation, values represent the average volume per pupa, determined by measuring volumes of 20 pupae (*n* = 10 replicates (vials) in GLU, *n* = 7 replicates in GAL, with 20 pupae per replicate). **(C)** Pupal weight after pupae formation, values represent the average weight per pupa, determined by measuring the total weight of 24-25 pupae, and dividing the total by the number of pupae (*n* = 10 replicates (vials) in GLU, *n* = 8 replicates in GAL, with 24-25 pupae per replicate). **(D)** Oxygen consumption rate of pupae, values represent the average oxygen consumption per pupa per min, determined by measuring the total consumption rate of 24-25 pupae, and dividing the total by the number of pupae (*n* = 10 replicates in GLU, *n* = 8 replicates in GAL, with 24-25 pupae per replicate). **(E)** Absolute oxygen consumption rate plotted against pupal weight. **(F)** Mitochondrial citrate synthase content in pupae as mitochondrial mass marker normalized by protein content (*n* = 8 replicates (vials) per group, with 5 pupae per replicate). Statistical analysis was performed by Student's *t*-test for panels B, C, D, and F and by ANCOVA for panel E (**P < 0.01; ****P < 0.0001).

The GAL pupal volume calculated from pupal length and width was significantly larger as was the pupal weight (Figures 2A - 2C). The oxygen consumption rate of the pupae was measured to estimate their energy expenditure. The oxygen consumption rate was significantly higher in GAL (Figure 2D). Since the GAL pupae showed higher body weight compared to GLU (Figure 2C), oxygen consumption was analyzed using an ANCOVA procedure (37, 38). Plotting the oxygen consumption rate against body weight showed no significant differences between the two groups (Figure 2E), indicating that the differences in oxygen consumption rate were body weight dependent. Nevertheless, normalized whole body mitochondrial mass appeared to be significantly lower in the GAL group (Figure 2F).

### 3.3 Nutritional programming effects on body weight and whole-body TAG at eclosion

**Figure 3:**
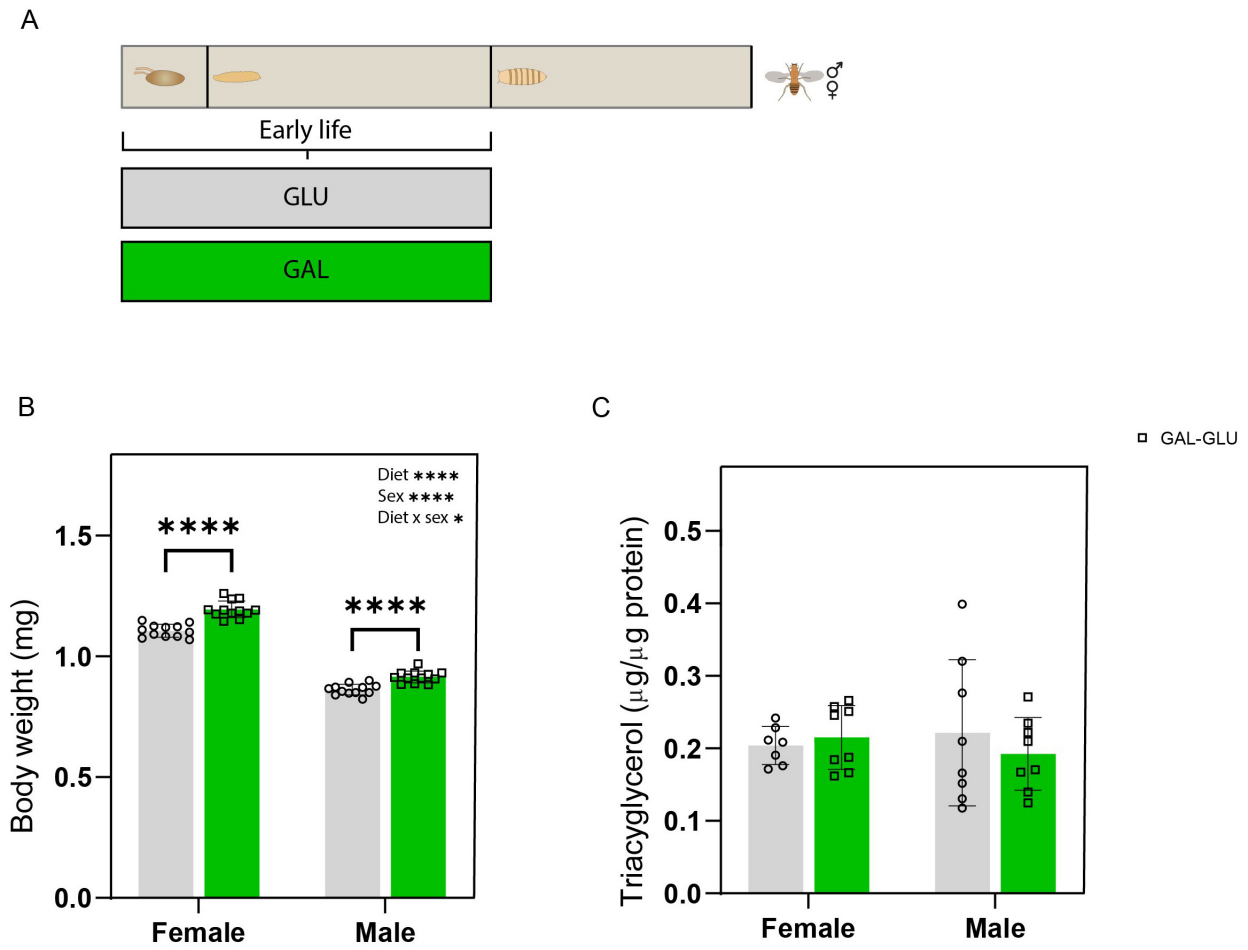
Galactose consumption in early-life (larval stage) increased body weight but had no effects on whole-body triacylglycerol (TAG) content of eclosed adult flies. **(A)** Experimental scheme. **(B)** Body weight of male and female flies after eclosion; values represent the average weight per fly per sex, determined by measuring the total weight of 20 male or female flies and dividing the total by the number of flies (*n* = 12 replicates (vials) per group, with 20 flies per replicate). **(C)** Whole-body TAG content of male and female flies after eclosion; TAG content was normalized by protein content (*n* = 8 replicates (vials) per group with 4 flies (males or females) per replicate). Statistical analysis was performed by two-way ANOVA using diet and sex as factors (*P < 0.05; ****P < 0.0001).

To investigate whether the early-life larval dietary interventions affected adult energy reserves, we continued the study, schematically shown in Figure 3A, focusing first on eclosion. The eclosed GAL male and female flies both showed significantly higher body weights, compared to GLU (Figure 3B). The interaction between the treatment and sex was significant, suggesting that larval galactose exerted sexually dimorphic effects on body weight at eclosion. The whole body TAG content was not significantly different between the two groups in both sexes (Figure 3C).

### 3.4 Nutritional programming effects on body weight, whole-body TAG, and lifespan upon feeding a standard glucose adult diet

**Figure 4:**
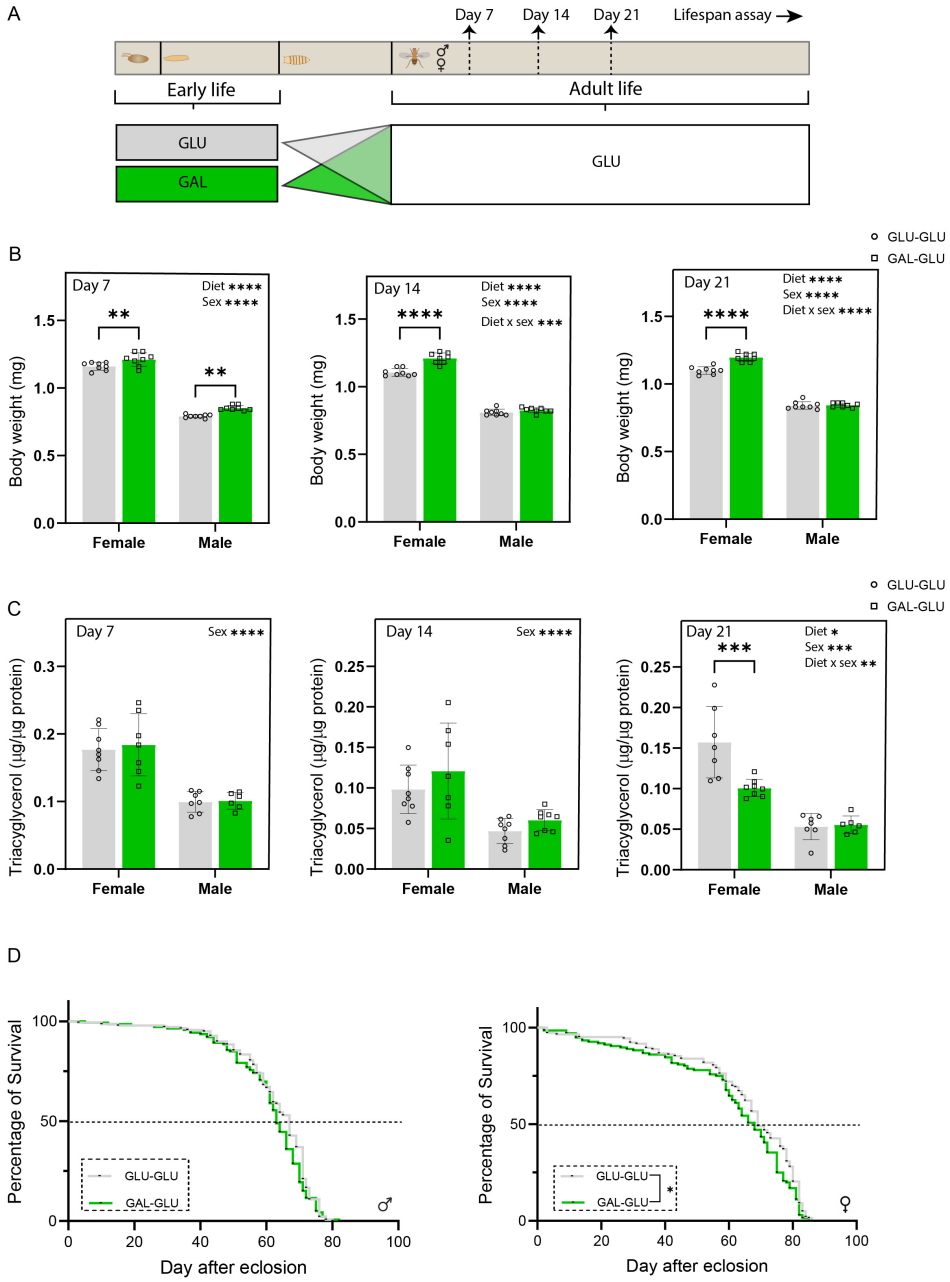
Galactose consumption in early-life (larval stage) had long-lasting effects in female, but not male. flies when maintained on the 5% glucose diet in later-life from eclosion onward. **(A)** Experimental scheme. **(B)** Body weight of male and female flies measured at 7, 14, and 21 days after eclosion, values represent the average weight per fly per sex at each timepoint, determined by measuring total weight of 10–20 male or female flies and dividing the total by the number of flies (*n* = 8 replicates (vials) per group, with 10–20 flies per replicate). **(C)** Whole-body triacylglycerol (TAG) content of male and female flies measured at 7, 14, and 21 days after eclosion, and TAG content was normalized by protein content (*n* = 8 replicates (vials) per group with 4 flies (males or females) per replicate). **(D)** Lifespan curves of male flies and female flies (*n* = 138 male flies in GLU, *n* = 139 male flies in GAL, *n* = 143 female flies in GLU, *n* = 136 female flies in GAL at start). TAG content at day 21 was analyzed by Generalized Linear Model. Statistical analysis was performed by two-way ANOVA using diet and sex as factors for other data in panels B and C. Survival curves in panel D were analyzed by the log-rank test (*P < 0.05, **P < 0.01, ***P < 0.001, ****P < 0.0001).

After sex-separation, the flies from either GLU or GAL were maintained on the same GLU diet during adult life (Figure 4A).

Both male and female flies from GAL-GLU showed significantly higher body weight at post-eclosion day 7, while the difference disappeared thereafter in males but remained in females (Figure 4B). The interaction between the treatment and sex was significant at days 14 and 21, suggesting sex specific effects.

Whole-body TAG content was not different between the two groups at all three time points in male flies, while it was significantly lower in the GAL-GLU group at post-eclosion day 21 in female flies (Figure 4C). The interaction between the treatment and sex was significant at day 21, suggesting sexual dimorphism.

The early-life diets did not show any significant programming effects on the lifespan curves in male flies (Figure 4D), nor on median survival, nor on maximum survival or mean lifespan (Table 2).

**Table 2:** Lifespan parameters.

Groups		GLU	GAL	GLU	GAL
**Early-life diets**		**5% glucose**	**5% galactose**	**5% glucose**	**5% galactose**
**Adult diets**		**5% glucose**		**20% glucose**	
Male	Median survival	67	63	71	71
	Maximum survival	76.6	76.4	80.6	80.1
	Average lifespan	61.2	61.9	65.2	65.7
	Log-rank test	0.09		0.78	
	Hazard ratios (GAL/GLU)	1.2 (95% CI: 0.95–1.5)		0.97 (95% CI: 0.77–1.23)	
Female	Median survival	69	68	64	63
	Maximum survival	83.8	82.6	78.5	79.4
	Average lifespan	65.1	61.4	57.1	56.9
	Log-rank test	0.02		0.86	
	Hazard ratios (GAL/GLU)	1.304 (95% CI: 1.03–1.65)		0.98 (95% CI: 0.75–1.28)	

Median survival, maximum survival, and average lifespan are given as days and values, and are calculated for the entire population within each experimental condition.

In contrast, in females, the early-life galactose diet had significant programming effects on the lifespan curves (Figure 4D). The median survival was only 1-day different, while the mean survival was 61.4 versus 65.1 days (GAL-GLU vs GLU-GLU, Table 2), with a Hazard Ratio of 1.304 (95% CI: 1.03–1.65) for GAL-GLU/GLU-GLU. Overall, this suggests that early-life galactose shows adverse programming effects on the lifespan in female flies.

### 3.5 Nutritional programming effects on body weight, whole-body TAG, and lifespan when fed an obesogenic adult diet

**Figure 5:**
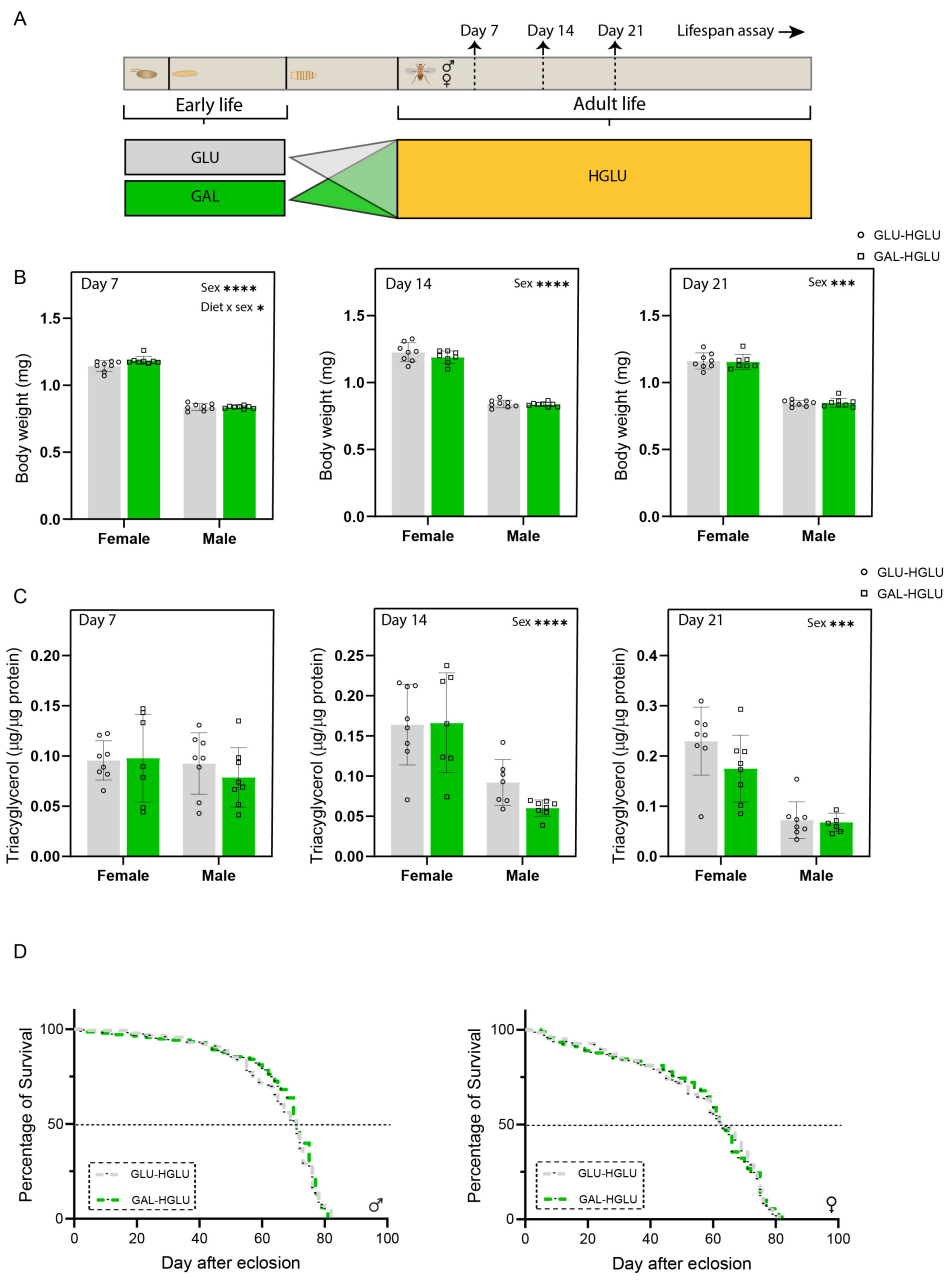
Galactose consumption in early-life (larval stage) did not have programming effects in flies maintained on the obesogenic 20% glucose diet in later-life from eclosion onward. **(A)** Experimental scheme. **(B)** Body weight of male and female flies measured at 7, 14, and 21 days after eclosion, values represent the average weight per fly per sex at each time point, determined by measuring total weight of 10–20 male or female flies and dividing the total by the number of flies (*n* = 8 replicates (vials) per group, with 10–20 flies per replicate). **(C)** Whole-body triacylglycerol (TAG) content of male and female flies measured at 7, 14, and 21 days after eclosion, and TAG content was normalized by protein content (*n* = 8 replicates (vials) per group with 4 flies (males or females) per replicate). **(D)** Lifespan curves of male and female flies (*n* = 141 male flies in GLU, *n* = 138 male flies in GAL, *n* = 123 female flies in GLU, *n* = 90 female flies in GAL at start). TAG content and BW at day 21 were analyzed by Generalized Linear Model. Statistical analysis was performed by two-way ANOVA using diet and sex as factors for other data in panels B and C. Survival curves in panel D were analyzed by the log-rank test (*P < 0.05, ***P < 0.001, ****P < 0.0001).

We then explored whether early-life dietary interventions show programming effects from eclosion onward in an obesogenic environment (Figure 5A).

After being challenged by a HGLU diet, the body weight (Figures 5B) and whole-body TAG (Figure 5C) at all time points, and lifespan curves (Figure 5D), were all not significantly different in both male and female flies.

## 4 Discussion

Our study demonstrates that early-life galactose exposure in *Drosophila melanogaster* exerts nutritional programming effects. In early-life, galactose intake prolonged developmental time, increased eclosion weight, and reduced mitochondrial mass. Crucially, these early-life effects translated into sex-specific effects on adult body composition and longevity. Galactose-fed females, but not males displayed a higher body weight but reduced TAG stores in adult life. In addition, these effects were only evident under standard dietary conditions (5% glucose) and were masked by an obesogenic diet (20% glucose), suggesting that the programming effects of galactose are sensitive to the adult nutritional environment.

Galactose is a highly relevant nutritional monosaccharide (as part of the milk sugar lactose), especially in mammals (39). Recently, galactose was also suggested to be a physiologically relevant monosaccharide in *Drosophila* (40), which is able to catabolize galactose (31). However, the metabolic effects of dietary galactose in *Drosophila* are still largely unstudied. This study is the first to show that dietary galactose versus glucose extended the developmental time during the larval period and increased pupal volume and weight, without affecting larval survival rate. These effects were unlikely to be induced by a calorie restriction-mimicking effect since calorie restriction usually leads to increased development time with decreased weight (41, 42), while we observed increased weight and volume. Critically, while glucose restriction (0.1% w/v vs. 5% w/v) reduces body weight without affecting development time (43), our galactose diet (5% w/v vs. 5% w/v glucose) simultaneously extended development and increased body weight. This suggests galactose exerts effects distinct from mere glucose insufficiency. In cellular metabolism, galactose is converted to glucose-1-phosphate via the Leloir pathway, and 1 ATP is consumed during this process. Glucose-1-phosphate is isomerized into glucose-6-phosphate, an intermediate of glycolysis. Evidence from *in vitro* studies suggests that galactose metabolism may increase mitochondrial respiration. For example, human cancer cells cultured *in vitro* in galactose-based media showed increased mitochondrial oxygen consumption versus those cultured in the glucose-based media (44). Similarly, human primary muscle cells (45) and bovine primary aortic endothelial cells (46) showed increased mitochondrial oxygen consumption in galactose-based media as well. Of note, in these *in vitro* studies, the mitochondrial mass was unaffected. Meanwhile, in a mouse model, dietary galactose did not affect body weight and whole body oxygen consumption but increased transcripts encoding intestinal oxidative phosphorylation (OXPHOS) components without affecting mitochondrial mass (47). Here, we observed that galactose versus glucose feeding of *Drosophila* larvae resulted in a lower mitochondrial mass *in vivo*. This suggests that galactose may have mitochondrial metabolic consequences *in vivo* that might be absent *in vitro*, such as altered mitophagy, mitochondrial biogenesis, or tissue-specific responses. The ANCOVA analysis suggests that the difference in pupal oxygen consumption between the two groups was body-weight-dependent, based on the method published before (38). Nevertheless, since mitochondrial mass was lower in GAL, we speculate that larval galactose consumption increases mitochondrial OXPHOS activity in the pupae. Moreover, we hypothesize that the extended developmental time might be due to reduced systemic insulin signaling, potentially mediated by mitochondrial-driven changes, as galactose is a less potent insulin secretagogue compared to glucose (48). In support of this, it has been shown that a lower mitochondrial OXPHOS activity, specifically in the fat body, was sufficient to shorten the larval developmental time in fruit flies via decreased fat-body expression of the negative regulators of insulin signaling *Eiger* and *ImpL2*, promoting systemic insulin signaling (49). A further speculation is that galactose mitigates systemic insulin signaling and increased mitochondrial activity might translate into the decreased TAG content in GAL-GLU-fed adult females. Nevertheless, given the observations in the different models, the effects of dietary galactose on development and mitochondrial activity deserve further investigation.

Our results do not support the hypothesis that early-life exposure to galactose exerts beneficial programming effects in *Drosophila* as those observed in mouse studies (22, 23, 50). Of note, in those mouse studies, the postweaning glucose or maltodextrin was partly, not fully, replaced by galactose. Interestingly, co-consumption of galactose and glucose, mimicking hydrolyzed lactose, significantly extends female lifespan in *Drosophila* (51). These findings suggest that not only the presence, but also the ratio in which dietary glucose and galactose are present, might have unique outcomes which need to be further investigated. Previously, it has been shown that life-long intervention with 6.5% of galactose induced a shortened lifespan in both male and female *Drosophila* (52), while we showed that 5% of galactose only in the larval period is sufficient to induce shortened lifespan in adult female *Drosophila* when a 5% glucose diet was provided in later-life. This implies that the larval period is a critical window for later-life health in *Drosophila*, and this agrees with the concept of developmental origins of health and disease (53). Our results may also support the predictive adaptive response hypothesis that the health condition is maximized if the early-life environment matches later-life environment (54).

After eclosion, both male and female flies from GAL had higher body weights, however, there was sexual dimorphism seen in the programming effects on the later-life body weight, whole-body TAG and lifespan since these effects were only observed in female flies, not in males. The observed sex-specific effects are consistent with other nutritional programming studies, although it depends on the intervention and the phenotype outcomes (55). It seems that females show higher susceptibility to the long-term metabolic effects of early-life nutrition (55). For instance, epidemiological data show that exposure to the Dutch famine during gestation increased later-life offspring's body mass index (56) and adiposity (57) only in women, not in men. Limiting the amount of carbohydrates in early-life of women (versus men) has more protective effects on type 2 diabetes development (12). This might be due to different responses to glucose between females and males; for example, women handle glucose changes differently than men (58), and the lifespan of female *Drosophila* is more susceptible to the detrimental effects of high sugar diets (59). Previously, we also observed sexual dimorphism in mice with galactose-induced programming effects on attenuated obesity seen only in female but not in male mice (22). This suggests that the nutritional programming effects of galactose show parallel sexual dimorphism in two different animal model systems. It is still not well understood why females are more susceptible to the galactose intervention, but it might be related to the sensitivity of female reproductive organs to energy status (60) and galactose metabolism (61).

Moreover, mouse studies also showed that the nutritional programming effects of galactose on attenuated obesity (seen when a healthier later-life diet was provided) were diminished when female mice were fed a more adverse obesogenic later-life diet (22, 23). The current *Drosophila* data showed that after being challenged by a 20% glucose obesogenic diet in later-life, programming effects on body weight, whole-body TAG content, and lifespan were absent in both sexes. This suggests that a healthier later-life diet would enhance the galactose-induced programming effects. However, this needs to be tested in the future.

Finally, we acknowledge several limitations in the present study. Our primary aim is to extend the galactose programming studies to the *Drosophila* model, and our physiological and survival data indicate a clear programming effect of early-life galactose. However, future studies employing metabolomics (to quantify body composition changes, e.g., protein content, glycogen, glucose, and trehalose), transcriptomics (to assess insulin signaling pathway gene expression) or mitochondrial assays (to measure, e.g., the oxygen consumption across lifespan) would pinpoint the exact molecular drivers of these metabolic changes. Another limitation of the current study is the fact that only one dietary dose of galactose was used, and it remains to be investigated whether other amounts (potentially in combination with other carbohydrates) will give dose-dependent responses or a similar response. The study clearly shows that programming effects of early-life galactose consumption were sex-specific and were subject to the later-life diet, but the molecular mechanisms leading to these outcomes need comprehensive investigation to fully understand the biological implications of our results. Additionally, females and males were not separated during the larval and pupal stages, and the sex ratio at eclosion was not quantified, excluding analysis of sex-specific (developmental) effects by these diets in early-life. Furthermore, we did not quantify female fecundity. Given that egg production costs energy in females, the reduction in TAG content in GAL-fed females might reflect a shift in resource allocation toward reproduction, which warrants future investigation.

Conclusively, we provide the first evidence that dietary galactose had significant effects on the larval developmental timing and gave sexual dimorphism in its nutritional programming effects in *Drosophila*, dependent on later-life (non)obesogenic diet composition.

## Data Availability

The raw data supporting the conclusions of this article will be made available by the authors, without undue reservation.
